# Electrical muscle stimulation prevents critical illness polyneuromyopathy: a randomized parallel intervention trial

**DOI:** 10.1186/cc8987

**Published:** 2010-04-28

**Authors:** Christina Routsi, Vasiliki Gerovasili, Ioannis Vasileiadis, Eleftherios Karatzanos, Theodore Pitsolis, Elli Tripodaki, Vasiliki Markaki, Dimitrios Zervakis, Serafim Nanas

**Affiliations:** 1 First Critical Care Department, National and Kapodistrian University of Athens Evangelismos Hospital, Ypsilantou 45-47, 106 75, Athens, Greece

## Abstract

**Introduction:**

Critical illness polyneuromyopathy (CIPNM) is a common complication of critical illness presenting with muscle weakness and is associated with increased duration of mechanical ventilation and weaning period. No preventive tool and no specific treatment have been proposed so far for CIPNM. Electrical muscle stimulation (EMS) has been shown to be beneficial in patients with severe chronic heart failure and chronic obstructive pulmonary disease. Aim of our study was to assess the efficacy of EMS in preventing CIPNM in critically ill patients.

**Methods:**

One hundred and forty consecutive critically ill patients with an APACHE II score ≥ 13 were randomly assigned after stratification to the EMS group (n = 68) (age:61 ± 19 years) (APACHE II:18 ± 4, SOFA:9 ± 3) or to the control group (n = 72) (age:58 ± 18 years) (APACHE II:18 ± 5, SOFA:9 ± 3). Patients of the EMS group received daily EMS sessions. CIPNM was diagnosed clinically with the medical research council (MRC) scale for muscle strength (maximum score 60, <48/60 cut off for diagnosis) by two unblinded independent investigators. Duration of weaning from mechanical ventilation and intensive care unit (ICU) stay were recorded.

**Results:**

Fifty two patients could be finally evaluated with MRC; 24 in the EMS group and 28 in the control group. CIPNM was diagnosed in 3 patients in the EMS group as compared to 11 patients in the control group (OR = 0.22; CI: 0.05 to 0.92, *P* = 0.04). The MRC score was significantly higher in patients of the EMS group as compared to the control group [58 (33 to 60) vs. 52 (2 to 60) respectively, median (range), *P* = 0.04). The weaning period was statistically significantly shorter in patients of the EMS group vs. the control group [1 (0 to 10) days vs. 3 (0 to 44) days, respectively, median (range), *P* = 0.003].

**Conclusions:**

This study suggests that daily EMS sessions prevent the development of CIPNM in critically ill patients and also result in shorter duration of weaning. Further studies should evaluate which patients benefit more from EMS and explore the EMS characteristics most appropriate for preventing CIPNM.

**Trial Registration Number:**

ClinicalTrials.gov NCT00882830

## Introduction

Critical illness polyneuromyopathy (CIPNM) is an acquired neuromuscular disorder observed in survivors of acute critical illness. It is characterized by profound muscle weakness and diminished or absent deep tendon reflexes [1] and is associated with delayed weaning from mechanical ventilation [2] suggesting a possible relation between limb and respiratory neuromuscular involvement. In addition, the syndrome is associated with prolonged hospitalization and increased mortality [3]. The diagnosis of CIPNM requires a reliable bedside muscle strength examination and depends on patient's cooperation and maximal effort [4].

Several risk factors have been identified including systemic inflammatory response and sepsis [5], medications such as corticosteroids [6] and neuromuscular blocking agents [7], inadequate glycemic control [8], protracted immobility [4], hypoalbuminemia [9], Gram-negative bacteremia [9] and severity of organ dysfunction [10]. Thus, looking for the potentially reversible risk factors and subsequent adjustment of therapy are so far advocated as preventive measures to decrease the risk of CIPNM.

Electrical muscle stimulation (EMS) has been used as an alternative to active exercise in patients with chronic heart failure (CHF) [11] and chronic obstructive pulmonary disease (COPD) [12,13]. Many of these patients, even those who are clinically stable, experience severe dyspnea on exertion, which can prohibit the regular application of conventional exercise training, considered necessary for an integrated therapeutic approach. In a recent systematic review, EMS implementation in most of the selected controlled clinical trials produced significant improvements in muscle strength, exercise capacity and disease-specific health status [14]. Recently, we identified an acute systemic effect exerted by EMS on peripheral microcirculation of critically ill patients [15]. Specifically, after performing a 45-minute session of EMS on the lower extremities, an improvement in the microcirculation of the thenar muscle as assessed by near infrared spectroscopy technique was observed.

Therefore, we hypothesized that EMS could beneficially affect muscle functional status in the critically ill. The scope of the present study is to assess the efficacy of EMS in preventing CIPNM in critically ill patients hospitalized in a multidisciplinary ICU. The primary endpoint was the diagnosis of CIPNM as assessed with the Medical Research Council (MRC) scale for muscle strength and secondary endpoints were the duration of weaning from mechanical ventilation and the ICU length of stay. Some of the results of this study have been previously reported in the form of an abstract [16].

## Materials and methods

### Patients

The study was approved by the Scientific Council and the Ethics Committee of the Evangelismos Hospital, Athens, Greece. Written informed consent was given by family members of all the patients included in the study.

All patients consecutively admitted to the multidisciplinary 28-bed university ICU of Evangelismos hospital, a 1000-bed tertiary care medical center, during the study period (September 2007 to June 2009) were prospectively considered for inclusion in the study. Exclusion criteria were: age under 18 years, pregnancy, obesity (body mass index >35 kg/m^2^), preexisting neuromuscular disease (e.g. myasthenia Gravis, Guillain-Barré disease), diseases with systemic vascular involvement such as systemic lupus erythematosus, technical obstacles that did not allow the implementation of EMS such as bone fractures or skin lesions (e.g. burns) and end-stage malignancy. Patients with cardiac pacemakers were also excluded from the study. Patients with the diagnosis of brain death were not considered for inclusion in the study. The Sequential Organ Failure Assessment (SOFA) [17], Acute Physiology and Chronic Health Evaluation (APACHE) II [18] and Simplified Acute Physiology (SAPS) III [19] severity scores were calculated for all patients on the day of admission. These scores have been developed for the assessment of disease severity and have a prognostic value in patients admitted to the ICU.

### Study design and randomization

On the second day after admission (24 to 48 hours after admission) patients with an APACHE II admission score of 13 or more were randomized after stratification in the intervention group (EMS group) or the control group. Stratification was made on age (50 years or younger or older than 50 years, which is the mean value of our ICU patient's age) and gender (male or female). Patients were thus distributed to one of four groups after stratification. Patients with an odd number (in each of these four groups) were assigned to the EMS group and patients with an even number were assigned to the control group. Patients assigned to the EMS group received daily EMS sessions of both lower extremities starting from the second day after admission until ICU discharge. Patients in the control group did not receive sham EMS.

### Electrical muscle stimulation

EMS was implemented simultaneously on the vastus lateralis, vastus medialis and peroneous longus muscles of both lower extremities. Patients received daily sessions. In the case of hairy skin, the skin was carefully shaven before the application. After shaving and skin cleaning, rectangular electrodes (90 × 50 mm) were placed on the motor points of the vastus lateralis, vastus medialis and peroneus longus muscles of both legs. The stimulator (Rehab 4 Pro, CEFAR Medical AB, Malmö, Sweden) delivered biphasic, symmetric impulses of 45 Hz, 400 μsec pulse duration, 12 seconds on (including 0.8 second rise time and 0.8 second fall time) and 6 seconds off, at intensities able to cause visible contractions. In case of doubt, contraction was confirmed by palpation of the muscles involved. The duration of the session was 55 minutes including 5 minutes for warm up and 5 minutes for recovery. EMS sessions were continued until ICU discharge.

### MRC muscle strength scale

The MRC score for clinical assessment of muscle strength was used for the diagnosis of CIPNM [2]. After interruption of sedation, patients were screened daily for awakening and comprehension until ICU discharge. MRC was assessed on the day the patients had a level of consciousness adequate to respond to at least three of the following orders ('open/close your eyes', 'look at me', 'put out your tongue', 'nod your head', 'raise your eyebrows') [20]. Three muscle groups in all four limbs were assessed with the MRC scale with values ranging from 0 (quadriplegia) to 60 (normal muscle strength). The following functions were assessed: wrist flexion, forearm flexion, shoulder abduction, ankle dorsiflexion, knee extension, and hip flexion [21]. Patients with an MRC score of less than 48 of 60 were diagnosed with CIPNM. The cut-off limit of 48 for the MRC score was selected because it indicates clinically significant weakness and has been used previously for the clinical identification of ICU-acquired paresis [2,22]. The MRC score was performed by two independent investigators that were familiar with this technique and no more than 24 hours elapsed between the two measurements. The two investigators were unaware of each others assessment and they provided written MRC scoring for each muscle group that was assessed. The mean value of the MRC score of the two investigators was used for the diagnosis of CIPNM. The investigators were not blinded as to patients' allocation.

### Weaning from mechanical ventilation

The weaning period was defined as the time from the onset of weaning trials until the day there was no need for mechanical ventilation for the next 48 hours (short-term weaning period). During the weaning process the patients underwent trials of spontaneous breathing on a T-piece.

Although weaning is usually defined as successful when there is no need for mechanical ventilation for the next 48 hours after extubation, long-term mechanically ventilated patients especially those with CIPNM often require reintubation and further mechanical ventilation for more than 48 hours after a successful short-term weaning. Therefore, we also assessed the time from the onset of weaning trials until the day after which there was no more need for mechanical ventilation until ICU discharge (long-term weaning period) [20]. Long-term weaning success was defined as the patient's ability to sustain spontaneous breathing until ICU discharge.

Finally, the duration of mechanical ventilation, the need for reintubation and the number of days off the ventilator were also recorded.

### Statistical analysis

Power analysis was performed prior to the study initiation. We calculated that to provide 80% power with a *P *value of less than 0.05 judged to be significant, a sample size of 44 patients would be needed to detect a difference of 25% in the incidence of CIPNM, which was found to be 37.5% in a previous epidemiological study from our ICU [9]. Analysis of patient data was by intention to treat. Patients randomized to the EMS group that did not finally receive any EMS sessions were not included in the analysis. The binary logistic regression, with and without adjustments, was used to compare the development of CIPNM between the EMS and the control groups. Normality of distribution was checked by employing Kolmogorof-Smirnof test. Baseline characteristics of continuous variables of the EMS group and control group for the whole cohort, those finally evaluated and those not evaluated were compared by unpaired Student's t-test or Mann-Whitney *U *test, in the case of no normal distribution. Qualitative variables at baseline were compared by chi-squared test. A chi-squared test with Yates' correction was also employed to compare the number of patients evaluated with the number of patients not evaluated between the EMS and the control groups. The Kaplan-Meier method was used to compare the duration of weaning between patients with and without CIPNM as well as between patients assigned to the EMS and control groups. The two groups were compared using the log-rank test. All continuous variables are presented by mean ± standard deviation (SD) or median and range in case of no normal distribution. The odds ratio (OR) and the 95% confidence interval (CI) are also reported in relation to the binary logistic regression. *P *values of less than 0.05 were considered statistically significant.

## Results

### Study population and randomization

**Figure 1:**
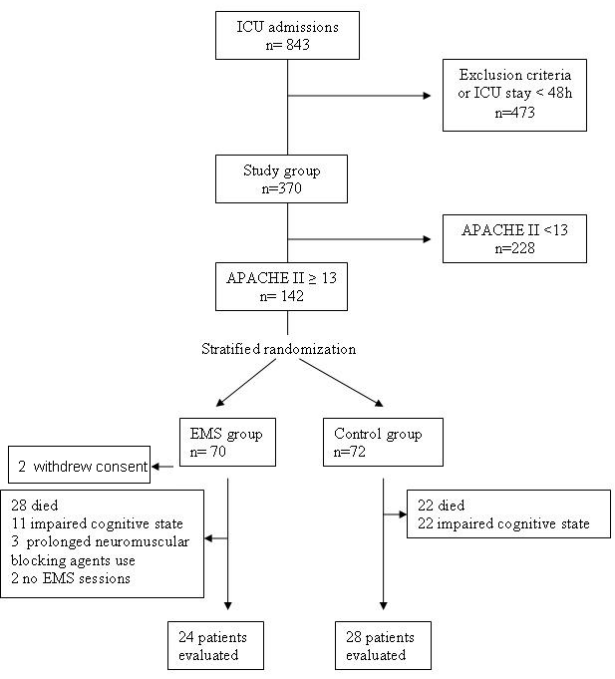
**Schediagram of patients admitted to the ICU and the randomization process**. APACHE, Acute Physiology and Chronic Health Evaluation; EMS, electrical muscle stimulation.

Eight hundred and forty-three patients were admitted to our multidisciplinary ICU during the 22-month study period and 471 patients fulfilled the exclusion criteria or had an ICU stay of less than 48 hours. In two patients, informed consent was not obtained in time for inclusion in the study and were, therefore, also excluded from the study. Of the remaining 370 patients, 142 had an APACHE II admission score of 13 or more and were thus considered for randomization. Of these patients, 70 were randomly assigned to the EMS group and 72 to the control group. Two patients in the EMS group withdrew their consent. Of the patients enrolled in the EMS group (n = 68), 28 patients died and 11 patients could not be evaluated with the MRC score due to impaired cognitive state. Five patients in the EMS group were excluded. The reason for exclusion was prolonged use of neuromuscular blocking agents (n = 3) and no EMS sessions during their ICU stay (n = 2). Of the patients enrolled in the control group (n = 72), 22 patients died and 22 patients could not be evaluated with the MRC score due to impaired cognitive state. Fifty-two patients were finally evaluated; 24 in the EMS group and 28 in the control group (Figure 1).

Baseline characteristics of patients randomly assigned to the EMS group or the control group are shown in Table 1. No statistically significant difference was found between the two groups in any of the variables.

**Table 1:** Baseline characteristics of 140 critically ill patients randomly assigned to the EMS group or the control group (mean ± standard deviation)

	EMS group (n = 68)	Control group (n = 72)
**Age, years**	61 ± 19	58 ± 18

**Gender M/F**	46/22	49/23

**Severity of illness at ICU admission**		
APACHE II score	18 ± 4	18 ± 5
SOFA score	9 ± 3	9 ± 3
SAPS III score	61 ± 13	60 ± 14

**Diagnostic category at admission**		
Sepsis/septic shock, n(%)	11 (16.2)	14 (19.4)
Trauma, n(%)	12 (17.6)	14 (19.4)
Post-surgical, n(%)	13 (19.1)	12 (16.7)
Brain injury, n(%)	24 (25.3)	23 (31.9)
Respiratory failure, n(%)	2 (2.9)	4 (5.6)
Other, n(%)	6 (8.8)	5 (6.9)

**Comorbidities**		
Cardiovascular, n(%)	31 (46)	34 (47)
Respiratory disease, n(%)	8 (12)	14 (19)
GI disease, n(%)	3 (4)	2 (3)
Hepatic disease, n(%)	3 (4)	1 (1)
Renal disease, n(%)	7 (10)	9 (13)
Diabetes melitus, n(%)	9 (13)	10 (14)
Hematological disease, n(%)	3 (4)	1 (1)
Other, n(%)	8 (12)	4 (6)
None reported, n(%)	17 (25)	23 (31)

**Duration of sedation, days**	12 ± 10	12 ± 11

**Sepsis/septic shock, n(%)**	54 (77)	58 (80)

**Aminoglycoside use, days**	5 ± 8	8 ± 11

**Corticosteroids use, days**	6 ± 9	5 ± 8

**Neuromuscular blocking agents use, days**	1 ± 2	3 ± 5

No statistical significant difference is noted between the groups in any of the parameters.

APACHE, Acute Physiology and Chronic Health Evaluation; EMS, electrical muscle stimulation; F, female; GI, gastrointestinal; M, male; SAPS, Simplified Acute Physiology Score; SOFA, Sequential Organ Failure Assessment.

Concerning the 52 patients who were finally evaluated, no significant difference was found between the EMS and control group in age (mean ± SD (range), 55 ± 20 (23 to 82) vs. 59 ± 21 (19 to 84) years, respectively, *P *= 0.41), gender (male/female, 19/5 vs. 22/6 respectively, *P *= 0.99), SOFA sore (mean ± SD (range), 8 ± 3 (3 to 13) vs. 8 ± 3 (3 to 16), respectively, *P *= 0.50) and SAPS III (mean ± SD (range), 55 ± 11 (40 to 82) vs. 58 ± 14 (34 to 95), respectively, *P *= 0.46). APACHE II score was statistically significant (mean ± SD (range), 16 ± 4 (13 to 25) vs. 19 ± 5 (13 to 31), respectively, *P *= 0.03).

Concerning the patients not evaluated, no statistically significant difference was found between EMS and control group in gender (male/female, 23/16 vs. 27/17, respectively, *P *= 0.99), APACHE II score (mean ± SD (range), 19 ± 5 (13 to 35) vs. 18 ± 5 (13 to 36), respectively, *P *= 0.13), SOFA score (mean ± SD (range), 9 ± 4 (4 to 21) vs. 9 ± 3 (4 to 14), respectively, *P *= 0.82) and SAPS III (mean ± SD (range), 65 ± 13 (43 to 99) and 61 ± 14 (33 to 97), respectively, *P *= 0.16). Age was higher in the EMS group (mean ± SD (range), 65 ± 17 (22 to 85) vs. 58 ± 16 (20 to 89) years, respectively, *P *= 0.03).

No difference was observed between the number of patients evaluated with the number of patients not evaluated between the EMS and the control group (*P *> 0.99).

### Diagnosis of critical illness polyneuromyopathy

**Figure 2:**
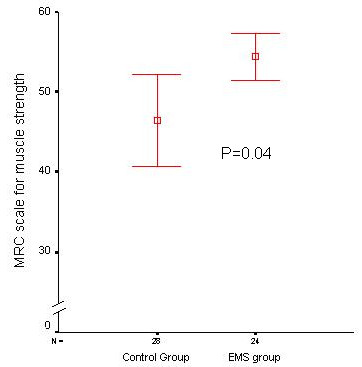
**Difference in the MRC scale for muscle strength between patients assigned to the EMS group as compared with patients assigned to the control group (mean ± 2 standard errors)**. *P *= 0.04. EMS, electrical muscle stimulation; MRC, Medical Research Council.

CIPNM was diagnosed in 3 patients in the EMS group as compared with 11 patients in the control group (OR = 0.22, 95% CI = 0.05 to 0.92, *P *= 0.04; Table 2). The incidence of CIPNM was also lower in the EMS group when comparison was adjusted for age and APACHE II score (OR = 0.22, 95% CI = 0.05 to 1.02, *P *= 0.05), variables for which a significant between-group difference was found in patients finally not evaluated and evaluated, respectively, according to the intention to treat approach. The MRC score was statistically significantly higher in patients assigned to the EMS group as compared with the control group (median = 58, range = 33 to 60 vs median = 52, range = 2 to 60, *P *= 0.04; Figure 2).

**Table 2:** Diagnosis of CIPNM in patients assigned to the EMS group as compared with patients assigned to the control group (*P *= 0.04)

	EMS group (n) (%)	Control group (n)(%)	Total
**CIPNM**	3 (12.5)	11 (39.3)	14

**No CIPNM**	21 (87.5)	17 (60.7)	38

**Total**	24	28	52

CIPNM, critical illness polyneuromyopathy; EMS, electrical muscle stimulation.

### Weaning from mechanical ventilation

The median short-term weaning period of critically ill patients was 2 (range = 0 to 99) days and the median long-term weaning period was 3 (range = 0 to 99) days. Four patients did not need mechanical ventilation during their ICU stay and one patient was not able to be weaned from the ventilator.

Patients that developed CIPNM had a longer short-term weaning period as compared with patients that did not develop CIPNM (median (range), 3 (0 to 44) vs. 1 (0 to 10) days, respectively, *P *= 0.003). Patients that developed CIPNM had a prolonged long-term weaning period compared with those without CIPNM (median (range), 12 (0 to 44) vs. 1 (0 to 10) days, respectively, *P *= 0.0001).

**Figure 3:**
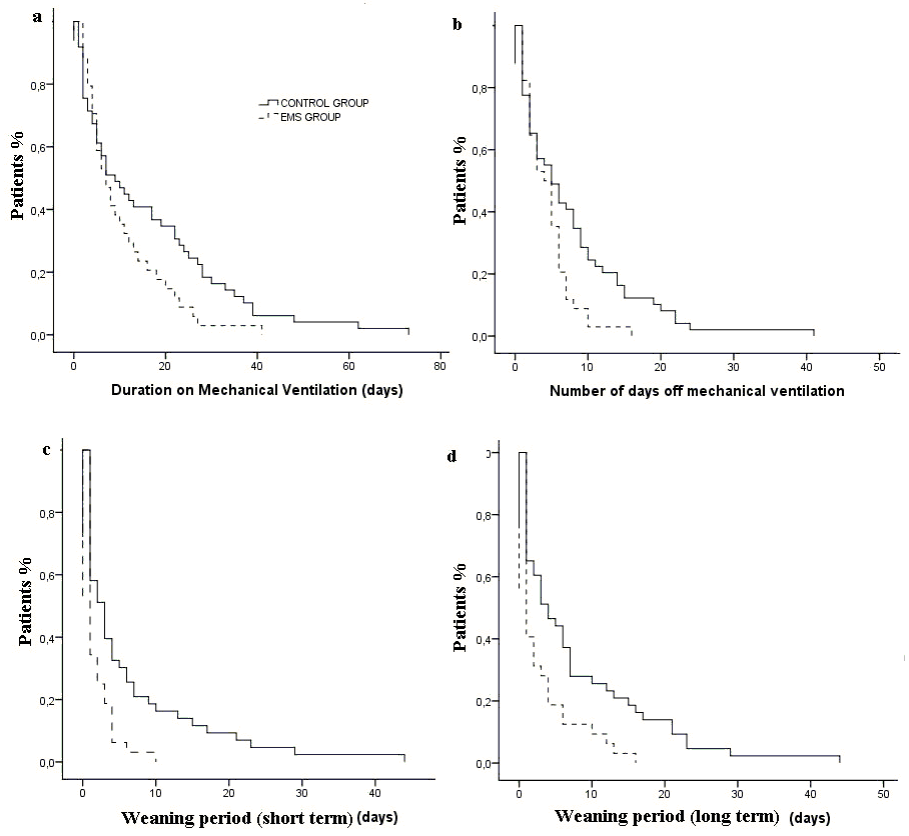
**Kaplan-Meier curves of the probability of remaining under mechanical ventilation after the onset of weaning**. **(a) **Duration of mechanical ventilation (log rank test, *P *= 0.075). **(b) **Days off mechanical ventilation (log rank test, *P *= 0.003). **(c) **Weaning period (short-term): no need for mechanical ventilation for the next 48 hours (log rank test, *P *= 0.003). **(d) **Weaning period (long-term): no need for mechanical ventilation until ICU discharge (log rank test, *P *= 0.003) for patients in the electrical muscle stimulation (EMS) group as compared with those in the control group.

The duration of mechanical ventilation was shorter for patients assigned to the EMS group compared with patients in the control group (median (range), 7 (2 to 41) vs. 10 (1 to 62), days, respectively), however, this difference was barely significant (log rank test, *P *= 0.07). The short-term weaning period was significantly shorter in patients assigned to the EMS group as compared with patients assigned to the control group (median (range), 1 (0 to 10) vs. 3 (0 to 44) days, respectively, log rank test, *P *= 0.003). The long-term weaning period was significantly shorter in patients assigned to the EMS group compared with those in the control group (median (range), 1 (0 to 16) vs. 4 (0 to 44) days, respectively, log rank test, *P *= 0.003). Finally, the number of days off the ventilator were significantly less in patients assigned to the EMS group compared with patients in the control group (median (range), 4 (0 to 16) vs. 6 (0 to 41) days, respectively, log rank test, *P *= 0.003; Figure 3).

Five patients (four in the control group and one in the EMS group) were reintubated.

### ICU length of stay

**Figure 4:**
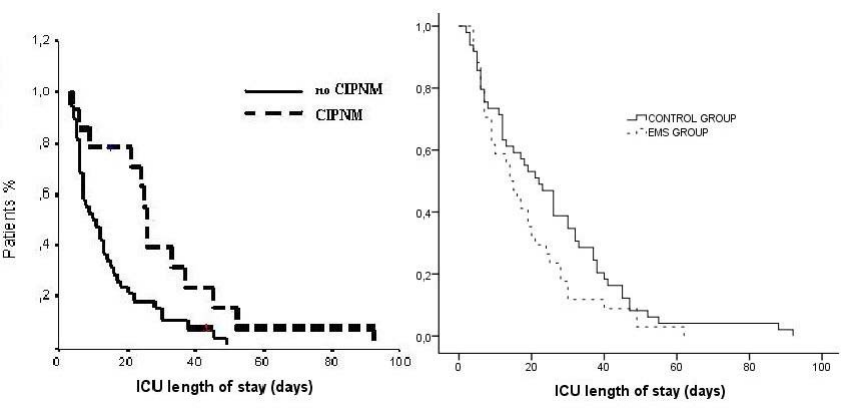
**Kaplan-Meier curves comparing the ICU length of stay in patients with and without critical illness polyneuromyopathy (CIPNM; log-rank test, *P *= 0.01) and between patients assigned to the electrical muscle stimulation group as compared with patients assigned to the control group (log-rank test, *P *= 0.11)**.

Patients that developed CIPNM had a longer ICU stay compared with patients that did not develop CIPNM (mean (range), 25 (4 to 92) vs. 11 (3 to 49), days, respectively, log rank test, *P *= 0.01). The duration of ICU stay, although shorter in patients assigned to the EMS group compared with those in the control group, was not found to be significantly different (mean (range), 14 (4 to 62) vs. 22 (2 to 92), days, respectively, log rank test, *P *= 0.11; Figure 4).

## Discussion

The main finding of our study is that daily, 55-minute EMS sessions prevented the development of CIPNM in critically ill patients. Furthermore, EMS treatment was associated with a shorter duration of weaning from mechanical ventilation.

For the prevention of CIPNM, avoidance of known risk factors, including hyperglycemia, is the only preventive measure proposed so far [22]. To our knowledge, this is the first randomized parallel intervention study to suggest that EMS can prevent the development of CIPNM in critically ill patients and preserve the muscle strength as assessed with the MRC score, thus potentially providing a preventive tool for this condition. The incidence of CIPNM was lower in the EMS group even after comparison was adjusted for variables for which a significant between group difference was found in baseline statistics in the subgroup of patients finally evaluated and not evaluated.

EMS has been extensively used as an alternative form of exercise in patients with severe COPD [12,13,23] and CHF [11]. In these patients who could not perform any form of active exercise due to respiratory or cardiac insufficiency, EMS was well tolerated and resulted in an improvement of muscle performance such as maximum voluntary contraction [13], muscle strength and endurance [12], and additionally, in an improvement of exercise capacity [11,13] and quality of life [11,23]. In a recent study involving bed-bound patients with COPD receiving mechanical ventilation after an ICU stay, EMS caused an increase in muscle strength, as assessed with the MRC scale, and reduced the number of days for transfer from bed to chair [23]. In a previous study by our group, daily EMS sessions implemented from the second day of ICU admission, to a large extent preserved the muscle mass of critically ill patients as assessed with ultrasonography [24]. Specifically, after eight days of daily EMS sessions on lower extremities (quadriceps and peroneus longus muscles) the decrease in muscle mass of the quadriceps muscle as assessed with ultrasonography was significantly less in the EMS group. In the present study, EMS was implemented in all patients assigned to the EMS group from the second day after admission with the only contraindication being the use of neuromuscular blocking agents. Three patients, although initially included in the study, were finally excluded due to prolonged use of neuromuscular blocking agents that did not allow the implementation of EMS. The rationale behind the choice of stimulated muscles was based on accessibility and functional significance for CIPNM. The daily EMS sessions were aimed at achieving the maximum possible effect. EMS was well tolerated and no side effects occurred during the sessions as has been previously described [15,24]. As it does not require the patient's cooperation it was easily applicable from the day of admission.

The pathophysiological mechanisms involved in this finding could be that EMS, as an alternative form of exercise, acts as an anabolic stimulus to the muscle reversing the catabolic effects of critical illness and immobilization. In an early study involving critically ill patients it has been shown that EMS has beneficial effects on muscle metabolism [25]. Addressing the pathophysiological bases of the effects of EMS on the main determinants of work capacity, Pérez and colleagues showed that EMS implementation in healthy young men improved oxygen uptake (VO_2_) kinetics and work efficiency [26]. Also, EMS applied to the lower limbs of critically ill patients induced an acute systemic effect on the microcirculation of the thenar as assessed with the near infrared spectroscopy technique, indicating the presence of factors induced by EMS, that act in a systemic way [15]. It is possible that molecules such as cytokines, produced at the loci of EMS and distributed via the circulation could be responsible for the systemic effect of EMS in preventing CIPNM observed in our study. Several cytokine levels, primarily IL-6 have been shown to increase after exercise [27,28]. IL-6 mRNA has been shown to increase after an EMS session in rat skeletal muscles [29,30]. Moreover, it is possible that central command and activation of metabo-reflex and/or ergo-reflex during EMS may increase sympathetic discharge and contribute to changes in heart rate, systolic blood pressure, blood volume and cardiac output, and therefore affect the skeletal muscle metabolism in a systemic way [31,32]. Finally, a bioenergetic pathway may be activated during EMS contributing to an improvement in mitochondrial function of the skeletal muscle [33]. Current approaches for CIPNM diagnosis comprise physical motor examination (e.g. with the MRC score for muscle strength) and electrophysiological investigations. The MRC score assessment depends on patient's cooperation and cannot be performed in patients who are not fully awake. Under these circumstances, CIPNM diagnosis relies on electrophysiologic studies [10,34]. However, several limitations exist and risks and costs of this testing should be taken into account. Conventional electrodiagnostic techniques often provide non-specific results or are hampered by local conditions (edema, multiple electrical devices) that prevent adequate disease classification [1]. Additionally, neurophysiologic testing results failed to predict duration of ICU stay or mechanical ventilation. Our main concern was to evaluate if EMS could affect the development of muscle weakness in our patients. Therefore physical examination has been selected as the primary determinant of CIPNM [4,20].

A number of studies have evaluated the role of early mobilization and/or physiotherapy in critically ill patients [35-40]. These studies involved passive limb mobilization [35-37], limb [35-38] and respiratory [37,39] muscle training, and bed cycling [40]. Although favorable results in terms of muscle strength [36-38], mobilization [37], six-minute walking distance [40] and ICU and hospital length of stay [35,37] have been shown, the development of CIPNM was not evaluated. It is also noteworthy that limb and respiratory muscle training require patient cooperation and could not be performed immediately after admission for the majority of critically ill patients, as opposed to EMS. Finally, intensive insulin therapy has been reported as a possible preventive tool for CIPNM [22].

A second finding from this study is that patients that developed CIPNM had prolonged duration of weaning process and prolonged ICU stay as compared with patients that did not develop CIPNM. This finding is consistent with the findings of recent studies that reported prolonged duration of weaning and prolonged ICU stay in critically ill patients with CIPNM as compared with patients without CIPNM [2,41].

Interestingly, patients assigned to the EMS group had a shorter duration of weaning as compared with patients assigned to the control group, which is further indication of the presence of a relation between limb and respiratory muscle weakness. As has been already mentioned an acute systemic effect has been reported after one EMS session [15]. It is possible that the reported systemic effect of EMS acts as an anabolic stimulus to the respiratory muscles as well. Generally speaking the procatabolic cytokine environment that characterizes disease states associated with inflammation and the critically ill, due to excessive localized elaboration of proinflammatory cytokines [42] may be altered by EMS. A possible role is suggested for IL-6, which reduces insulin-like growth factor 1 production, providing a major mechanism by which chronic inflammation inhibits hormonal anabolic action and affects growth [43]. Exercise training reduces IL-6 production as well as the magnitude of the acute exercise IL-6 response and a decreased plasma IL-6 concentration, not only in response to exercise but also at rest, appears to characterize a normal exercise adaptation [44]. The same mechanism, affecting muscle protein turnover, could hold true for EMS implementation. The shorter duration of weaning in patients assigned to the EMS group implies a beneficial effect of EMS on respiratory muscle function and reinforces the clinical significance of this study.

### Clinical implication

This is the first randomized parallel intervention trial to suggest that EMS of lower extremities can prevent the development of CIPNM in critically ill ICU patients. Daily EMS sessions prevented the development of CIPNM, preserved the muscle strength and shortened the duration of weaning and ICU stay in critically ill patients. EMS is an easily applicable, well-tolerated form of exercise that does not require patient cooperation, can be applied in any muscle group and can be implemented immediately after ICU admission. In our study, daily EMS sessions prevented CIPNM and shortened the duration of weaning from the ventilator, thus contributing to decreased morbidity and shorter ICU stay.

Further studies are needed to define which patients would benefit most from this intervention and to explore the EMS characteristics (current characteristics and muscle groups) that are most appropriate for preventing CIPNM.

### Limitations

The main limitation of our study is the relatively small number of critically ill patients that were able to be evaluated for the development of CIPNM. In addition, we did not use sham-EMS sessions in patients assigned to the control group. Furthermore, the investigators performing the MRC scale were not blinded to patients treatment assignment (EMS or control). However, they were unaware of each others' assessments. In addition, the effect of EMS was assessed on muscle strength, but not on muscle function or ability to perform activities of daily living because our study was conducted in the ICU setting. Also, an electrophysiological evaluation was not performed, which would have assisted in the evaluation of patients who did not regain adequate cognitive function in order to perform the MRC scale. Finally, the high mortality rate due to the increased illness severity should be noted.

## Conclusions

CIPNM is a common complication of critical illness associated with muscle weakness and prolonged duration of weaning and ICU stay. This is the first randomized controlled study suggesting that daily EMS sessions can prevent the development of CIPNM in critically ill patients and also result in shorter duration of weaning, thus potentially providing a preventive tool for this condition.

## Key messages

• CIPNM is a common complication of critical illness presenting with muscle weakness, areflexia, prolonged duration of weaning from mechanical ventilation and prolonged ICU and hospital stay.

• Searching for the potentially reversible risk factors and subsequent adjustment of therapy are so far advocated as preventive measures to decrease the risk of CIPNM.

• EMS is a form of exercise that does not require patient cooperation and has been used in patients with severe COPD and CHF. Previous studies from our group have suggested that EMS has an acute systemic effect and preserves the muscle mass of critically ill patients.

• This is the first randomized parallel intervention study to show that EMS can prevent the development of CIPNM in critically ill patients, thus effectively providing a preventive tool for this condition.

• EMS sessions also resulted in significantly shorter duration of weaning period from the ventilator.

## Abbreviations

APACHE: Acute Physiology and Chronic Health Evaluation; CHF: chronic heart failure; CIPNM: critical illness polyneuromyopathy; COPD: Chronic Obstructive Pulmonary Disease; EMS: electrical muscle stimulation; IL-6: interleukin-6; MRC: Medical Research Council; OR: odds ratio; SAPS: Simplified Acute Physiology; SD: standard deviation; SOFA: Sequential Organ Failure Assessment.

## Competing interests

The authors declare that they have no competing interests.

## Authors' contributions

All authors have contributed substantially to the submitted work and have read and approved the final manuscript. In particular CR participated in the design of the study, data analysis, drafting of the manuscript and revised critically the manuscript. VG participated in the design of the study, data acquisition, analysis and drafting of the manuscript. IV, LK, ET, DZ and TP participated in data acquisition, analysis and drafting of the manuscript. VM revised critically the manuscript and contributed to data analysis. Finally, SN conceived of and helped with the coordination of the study, revised critically the manuscript and provided final approval.
